# Study on the Interaction Effect between the Intensity of Government Low-Carbon Subsidies and the Growth Ability of Green and Low-Carbon Emerging Enterprises

**DOI:** 10.3390/ijerph20032438

**Published:** 2023-01-30

**Authors:** Lixia Chen, Jianyuan Huang

**Affiliations:** 1 School of Marxism, Hefei Normal University, Hefei 230601, China; 2 School of Public Administration, Hohai University, Nanjing 210098, China

**Keywords:** government low-carbon subsidies, green and low-carbon emerging enterprises, ability to grow, effect of interaction

## Abstract

With the development of science and technology and society, people’s demand for a healthy living environment is increasing, and the expression “low carbon” has become a daily feature of people’s lives. The emergence of a low-carbon economy, the impact on the traditional industrial structure and the formation of a new economic landscape make China, a developing country, eager to seize this opportunity to enhance its international competitiveness. To achieve this, it is necessary to establish a low-carbon concept, to actively restructure industrial and develop low-carbon industries; only in this way can we take advantage of the new round of industrial restructuring and grasp the initiative of development. Therefore, this paper selects data from enterprises in the emerging low-carbon industry, and uses a SVAR model to conduct a dynamic interaction analysis between government subsidy intensity, enterprise profitability, asset growth capacity and enterprise size. The results of the study show that the intensity of government subsidies in the first period has a certain positive effect on a company’s current profitability and asset growth, the improvement being most significant on its profitability. Among the larger companies, asset growth and profitability in the first period had a significant positive impact on current earnings, and the contribution of profitability to the company’s own performance was much larger than the average; among the smaller companies, asset growth and profitability in the first period had a significant positive impact on current earnings, and the contribution of asset growth to the company’s own performance was much larger than the average. The intensity of prior government subsidies in the Highs group has a significant positive relationship with the intensity of current government subsidies, the firm’s asset growth capacity and profitability; the intensity of prior government subsidies in the Lows group only has a significant positive impact on the asset growth capacity in the current period. In addition, the interaction between the three core variables in the Highs group is also significantly higher than that in the Lows group. This indicates that the government should implement differential policies and financial subsidies according to the actual needs of enterprises to maximize the effect of capital use and promote the development and growth of emerging enterprises.

## 1. Introduction

In the context of global warming, the “low-carbon economy” that advocates energy saving and low pollution has gradually become a hot topic around the world, and more and more companies are implementing low-carbon emission reductions [1]. With the development of the global economy, the emissions of carbon dioxide and other greenhouse gases are increasing year by year, leading to global warming which has a huge negative impact on the global ecosystem. At the same time, it also brings a series of negative impacts on the world economy [2,3]. As the global financial crisis caused by climate change has attracted increasing international attention, various countries have put forward the concepts of “green”, “low carbon” and “sustainable development” [4]. Our country has a large population, dependent mainly on the coal industry; in order to develop a low-carbon economy and promote the development of new industries, we must control the traditional coal and related industries, and develop the tertiary sector, notably tourism, and the service industry in order to reduce carbon dioxide emissions. Now that the goal of a low-carbon economy is increasingly attractive, and the development momentum is increasingly fierce, the future will definitely be a world of new companies, and low-carbon economies will become mainstream; we therefore need to do a good job developing new industries, especially high-tech ones, not least in the energy sector [5,6].

At present, the state has introduced a series of supportive policies to promote the development of emerging industries and strengthen support for the low-carbon economy. Compared with traditional industries, new enterprises pay more attention to the harmony between economy, society and human beings, which promotes the prosperity of a low-carbon economy, leads the development of the economy, paves the way for the development of society and lays a solid foundation for the development of society [7]. During the 13th Five-Year Plan period, green, low-carbon, new energy, new materials, green environmental protection and other emerging industries developed rapidly. The 14th Five-Year Plan proposed new energy, materials, environmental protection and other new technologies, accelerated the application of innovative technologies, and promoted the transformation of China’s economic structure under the framework of a carbon neutral strategy and the construction of an environmentally friendly society [8,9,10,11]. New, green, low-carbon industries are the key industrial support to achieve carbon neutrality, but also the locus of the contradiction between the continuous increase in total energy demand and the state-proposed energy saving and emission reduction [12]. The special characteristics of green and low-carbon industries require both a critical period of resource conversion in China and a greater scope for their development. Among industrial policies, government subsidies are a common policy instrument [13,14,15]. If developed in the direction of institutionalization and standardization in order to create a good start for the development of a low-carbon economy in China, they will bring the greatest benefits.

In the process of enterprise development and growth, the role played by traditional cost advantages is becoming less and less obvious. For new energy listed companies, they must have extremely strong R&D and innovation strength to be able to avoid homogenization of their own products with their peers and form core competitive advantages. In reality, the innovation achievements of enterprises are easily imitated or even acquired by competitors due to technology spillover, which reduce their economic benefits and weaken the innovation incentive effect. Insufficient innovation incentives demotivate R&D personnel, which limits the development and growth of enterprises. By means of subsidies, the government can mobilize enterprises’ R&D initiative. With the aim of an innovation-driven and low-carbon economy, the state has introduced a series of support policies to support the development of a new energy industry and encourage the R&D enthusiasm of new energy enterprises; moreover, those who have applied for state subsidies and passed the approval can obtain corresponding policy support, thus reducing R&D cost and R&D risk and attracting more enterprises to conduct technology R&D. However, in practice, the national subsidy policy is somewhat problematic and unscientific, and certain problems may arise: for instance, in order to enhance their performance, local governments may put a lot of money into the projects of certain high-tech companies, but the funds may be left idle by these companies, leading to overcapacity. Therefore, this paper explores the subsidy problem in the new energy industry, deeply analyzes the interactive relationship between the intensity of government subsidies and the growth capacity of enterprises, and analyzes the influence mechanism of government subsidies on the growth of enterprises, so as to provide a reference for the actual government subsidy policy formulation and implementation and thus promote the effective allocation and use of funds.

## 2. Literature Review

Wang (2022) believes that in today’s low-carbon development, in order to achieve sustainable development of Chinese enterprises, a strong low-carbon competitiveness must be established [16]. If we want to create a green and low-carbon development environment, we must invest heavily in this area. However, if spending is treated as a cost or an investment to reduce CO2 emissions, it leads to completely different behavioral and economic consequences [17]. In order to reduce carbon dioxide emissions and create an environmentally friendly industry, enterprises have to pay the price for the negative impact brought on by economic development. However, treating carbon emissions as a by-product of enterprises cannot fundamentally solve the problem of carbon emissions [18,19]. On the contrary, enterprise expenditure should be regarded as an investment to comprehensively change the enterprise’s strategic goals, value chain and business activities, integrate them into the enterprise’s production and operation process, and reconstruct the company’s value creation system through continuous innovation and continuous improvement [20,21,22,23]. The company should increase all kinds of inputs to reduce carbon emissions rather than reduce costs, so that it becomes a by-product of the company’s production and operation system, continuously improves the company’s low-carbon competitiveness, and maintains a competitive advantage in the future sustainable development [24,25,26]. In our country, enterprises should focus on management and innovation in the process of developing low-carbon energy. Low-carbon operation is a kind of operation improving the traditional business model to enhance the efficiency of resource utilization and reduce carbon emissions so as to achieve effective control of carbon and then improve the input and output of resources so as to optimize the allocation of enterprise resources. To strengthen the construction of low-carbon innovation capacity, we should not only increase the investment in innovation, but also fundamentally change the business concept of enterprises [27]. Under the restriction of “green and low-carbon” development, enterprises must change from the short-term concept of expense to the long-term concept of investment and development [28]. The change from the concept of cost to investment is the re-optimization of the enterprise’s production and operation system, and is a key way for enterprises to achieve low-carbon development, seek the growth of resources and capacity, and achieve low-carbon competitive advantages. Guided by the correct development concept and strategic goal, the company must carry out the reform of many aspects, such as system design, key resource investment and core capability building [29,30]. The results show that different industries and enterprises face different market environment and low-carbon competition pressure; their development power is different, and the same enterprises are also different in the direction and link of low-carbon development [31,32,33]. Therefore, if enterprises want to realize the transformation of low-carbon economy, they must flexibly choose the intensity and direction of implementation according to their own actual conditions.

Meng’s (2022) research shows that the characteristics of enterprises are closely related to their low-carbon practices, and the entrepreneurial characteristics, number of enterprises, ownership and regional differences of enterprises will all have an impact on them [34]. In the era of low-carbon economy, energy conservation and emission reduction are not only a burden, but also a strategic choice, which is the opportunity for the company to find new competitive advantages [35]. In the resource-based view, resources and capabilities are important factors for a company to obtain excess profits and sustainable competitive advantages. Therefore, a company will make corresponding strategic choices in investment, technology, environment and other aspects [36,37,38,39]. Enterprise size is an important index to measure the competitive advantage of enterprises, which is directly related to their strategic decision, leading to the integration and allocation of resources and to the selection of specific low-carbon policies [40]. At the same time, the resource-based view also emphasizes the company’s ability to improve the natural environment, and the low-carbon practice level is an important indicator for the company to improve its environmental quality [41,42,43]. Due to the constraints of resource endowment, technology level, production process and other factors, low-carbon practices in various industries differ greatly in terms of cost input and output benefits. Therefore, enterprises will adopt corresponding low-carbon strategies according to their own carbon emission reduction space and potential to achieve better emission reduction effects [44,45,46].

In the research of Wang (2022), it is asserted that in order to achieve the goal of “double carbon”, China is accelerating the pace of energy conservation, emission reduction, and zero carbon emission reduction [47]. In the era of low-carbon economy, the consumers’ tendency to choose low-carbon options is increasingly prominent. The production and sale of low-carbon products are completed through the low-carbon supply chain. In order to reduce carbon emissions, retailers also need to promote low-carbon publicity to increase people’s preference for low-carbon environment, so as to expand the market of low-carbon products [48,49]. At the same time, the government has rolled out a large number of financial subsidies to encourage manufacturing enterprises to carry out energy conservation research [50]. Therefore, it is of both theoretical and practical significance to discuss the collaborative emission reduction and energy-saving publicity decisions of enterprises in the supply chain under the condition that the government provides subsidies for manufacturers to reduce emissions [51,52,53].

In the research of Yang (2016), it is asserted that in order to realize low-carbon economy, it is necessary to achieve the goals of low carbon, low emission, low energy consumption, high cleanliness and low pollution [54]. Developing new industries can not only reduce carbon dioxide emissions, but also promote the development and application of new energy and reduce energy consumption and environmental pollution so as to better adapt to people’s high-quality life; in our traditional way of economic development, there exists a development mode that views environment and economic interest as a cost [55,56,57]. The exploitation and underutilization of coal, oil and natural gas have caused great damage to the natural environment and seriously polluted it [58,59,60,61]. The new low-carbon economy should completely change the previous plunder of resources and focus on the harmonious development of man and nature. New companies should strive for environmental protection, improve the utilization efficiency of resources, and create a recyclable, low-carbon and low-pollution development mode. In order for emerging industries to achieve better development, they should not only modify their foundation principles, but also have a good external environment [62]. Proactive policies and advanced technology are the most important factors, and to grasp this opportunity, it should be approached seriously. First, the emerging low-carbon economy should be actively supported by introducing, formulating and implementing proactive policies, such as increasing investment in emerging industries. Priority should be given to encouraging new industries that grow rapidly and improving the processing efficiency of emerging services [63,64,65,66]. In addition, relevant tax policies can be adjusted, for example, with the introduction of increased taxation of companies with high carbon dioxide emissions and the goal of providing economic incentives to companies with low emissions in the form of reducing their taxes, and so on [67]. Through the above policies and measures, carbon emission can be reduced, the development of new and low-carbon industries can be promoted, and these initiatives can also contribute to the transformation of the industry and technological progress [68]. Science and technology are the primary productive force behind the development of low-carbon industry; therefore, w the investment in science and technology and the development of low-carbon technology should be strengthened [69,70,71].

Some studies have shown that government grants and R&D investments work in both directions. By compensating for the loss on the company’s books, the government increases the company’s profitability and compensates for the externality of R&D costs that prevents the company from monopolizing R&D revenues, thus increasing the company’s enthusiasm for R&D investment [72]. However, if the size of government subsidies is too large, the company will have a “rent-seeking” mentality, which will reduce the company’s innovative behavior and thus negatively affect the company’s research development. Therefore, as government subsidies to companies increase in distribution frequency, the relationship between government and companies is no longer a simple linear relationship, having instead a three-fold effect. R&D projects of listed manufacturing companies require large amounts of capital and are more susceptible to financing constraints than other investments. Therefore, from the perspective of financing constraints, the impact of government subsidies on firms’ R&D investments can be divided into three levels. The first stage is characterized by the low government subsidies. All government subsidies promote research in firms with high and low financing constraints; however, firms with low financing constraints are less incentivized than those with high financing constraints. The second stage implies that as government grants to firms increase, R&D investment in firms with high and low levels of constraints continues to grow as government grants increase. In the third stage, as government subsidies continue to increase, their incentive effect on high and low financing constrained groups decreases. Therefore, increased government subsidies will cause firms within different financing constraints to form rent-seeking relationships with local governments with the aim to obtain better resources rather than relying on their own innovation and R&D to improve their competitiveness.

Some scholars have analyzed the effect of government subsidies on innovative SMEs in China, which has been extensively discussed by domestic scholars at present [73]. First, we discuss the positive effect of government subsidy policy. Government subsidies boost the development of a company and improve productivity, investment efficiency and innovation efficiency. The new economic growth theory points out that the development of innovative enterprises is characterized by the failure of the market, which inhibits the enthusiasm of R&D and then affects the development of enterprises, especially for innovative SMEs, so government subsidies can play a positive role. Second, we consider the negative effect of state financial subsidies. Government subsidies seriously affect the development of production and decrease the production efficiency and investment efficiency.

Through studying the relationship between corporate R&D investment and government subsidies [74], it has been shown that government subsidies can promote a company’s R&D investment, and the more STI subsidies a company receives, the higher its R&D investment. Through government subsidies, firms can be motivated in many ways to increase their investment in technological innovation, enhance their self-confidence, and thus promote their innovation output. To a certain extent, government subsidies can effectively suppress the negative effect of “market failure” and thus increase private enterprises’ R&D investment; by subsidizing enterprises’ research and innovation activities, subsidies can reduce the financial pressure of enterprises in research. By providing direct financing to enterprises, the government can encourage the companies to invest more in research and promote their research and technological innovation. Secondly, according to China’s tax law, the development of new technologies, new products and new processes is deducted at a rate of 15% on the basis of calculating taxable income. Therefore, with the tax benefits, subsidies can promote the innovation of enterprises and increase the R&D expenditure.

From the above literature analysis, it can be seen that government subsidies have different promotional effects on firms of different sizes and profitability, and the functions of government subsidies can vary significantly across the life cycle of a firm’s development, and many scholars have different views on the promotional effects of government subsidies on firm development. Some studies have pointed out that among the factors measuring firm growth, earnings and capital appreciation are an important factor in measuring firm development, reflecting the development status of the firm from several perspectives [75]. This paper empirically analyzes the interactive effects among the indicators based on the intensity of government subsidies, corporate profitability, and corporate asset growth using the PVAR model.

## 3. Research Design

### 3.1. Research Methods and Models

This paper uses panel vector autoregressive model (PVAR) as the main research method to objectively describe the dynamic and continuous interaction between enterprises and policies. PVAR is a system that takes variables as endogenous, and includes the lag terms of all variables into it so that it has practical significance. In the establishment of the model, there is no artificial presupposition of causality and no set of explained and explained variables, while the specific causal relationship and interaction conditions are judged by objective conclusions. The generalized moment estimation (GMM) model is established and solved. The overall form of PVAR model is shown in Formulas (1) and (2):

(1) $$y_{it}=\alpha_{0}+\sum_{j=1}^{p}\beta_{j}y_{it-j}+f_{i}+d_{t}+u_{it}$$

(2) $$y_{it}=\left\{\begin{array}{c} sub_{it}\\ Gti_{it}\\ EP_{it} \end{array}\right\}$$

In Formula (2), *y_it_* is the endogenous variable of the *i*-th listed company in year *t*, and the order is as follows: government subsidy intensity (Sub), green technology innovation (*Gti*), environmental performance (*EP*); *i* indicates the sample listed company. The value ranges from 1 to 132. *t* is the sampling selection interval from 2013 to 2021; *j* is the hysteresis order, *y_it−j_* is all endogenous variables, α_0_, β*_j_*, *f_i_*, *d_t_* and *u_it_* are intercept, regression coefficient matrix, fixed effect, time effect and random disturbance terms, respectively. In order to solve the PVAR model with all profile models having the same format (which is actually difficult to achieve), Love suggests using fixed-effect heterogeneity fi and employing a “positive mean difference” method to eliminate individual interests to avoid the deviation due to mean difference. At the same time, time effect dt is introduced to reflect its influence on time. In addition, taking the delay time as the instrumental variable, the generalized moment is used to estimate the short-term effect factor of GMM [76].

### 3.2. Indicator Selection

#### 3.2.1. Core Variables

The intensity of government subsidies, labeled Sub, are a key aspect for companies to reduce costs and increase profits. The main government financial subsidies are technology grants, government incentives, tax rebates, tax breaks, etc. Green technology innovation, labeled Gti, is an effective environmental management method that enhances the adaptability of companies to the environment and reduces the damage of innovation to the environment. Environmental Performance, labeled EP, is a specific action of a company aiming to invest in environmental management.

#### 3.2.2. Heterogeneous Grouping Variables

This paper groups the above core variables into heterogeneous grouping variables for the study. Firm size, labeled Aversize, describes the effects of government subsidies on firms of different sizes. A higher value of this ratio indicates that firms receive government subsidies with higher intensity. Asset growth, labeled Grow, is significantly lower for firms with higher asset growth than for firms with weaker asset growth, and this ratio indicates that firms with weaker asset growth are more likely to be subsidized. Profitability, labeled Nsale, is significantly lower for firms with higher profitability than for firms with weaker profitability, and this ratio indicates that firms with weaker profitability are more likely to be subsidized.

#### 3.2.3. Data Source and Processing

This paper selects new energy industries such as new energy, new materials and new energy vehicles as the important resources, removes ST, * ST and samples with missing data, and finally determines 150 enterprises. In this paper, data from 2013–2021 are selected, among which data from 2013 are only used to calculate the company’s growth capacity in 2014. Therefore, core variables from 2014 to 2021 is retained, with a total of 1200 observed values. The data used in this paper are obtained from Wind and CS-MAR databases and analyzed in the range of 1–99%. As shown in Table 1, the core variables are simply defined in this paper as the strength of free assets provided by the government to a specific company, while the company’s development potential, development trend and development speed refer to the company’s expansion capacity.

### 3.3. Descriptive Statistics

The descriptive statistics of variables are displayed in Table 2, which shows a result without indentation to more objectively represent the characteristics of the original data.

As can be seen from Table 2, the government subsidy intensity (Sub) of the Lows group is higher than that of the Highs, indicating that the policy is tilted toward the Lows group, which is consistent with previous research results. The growth capacity of the whole sample, Grow, is 0.392, indicating that the company’s development capacity is relatively stable in green, low-carbon and other emerging industries. Through the comparison of the maximum and minimum two indicators, it was determined that the minimum environmental performance (EP) value is 0.0001, and the average value is 0.0014, which indicates that enterprises do not pay enough attention to environmental governance, and the performance of environmental protection is generally not high. The average value of the green technology innovation (Gti) index is 17.13, indicating that the green technology innovation level of all companies improved on the whole. The maximum value, minimum value and standard deviation are 22.236, 0 and 4.842, respectively, so the research results show that there are significant differences in the level of green technology innovation of Chinese listed companies. From the perspective of profit, the profit of the Lows group is higher than that of the Highs, which indicates that the Lows group has relatively strong profitability in the emerging industry. This may be because smaller companies have stronger adaptability and respond more quickly to market changes.

Then, we analyzed the heterogeneity of asset growth and determined that, on average, the Highs group’s government funding strength Sub is significantly lower than that of the Lows group, indicating that Lows companies are more easily funded. From the perspective of environmental performance (EP), the Lows group has a better environmental performance. On an average Gti, the Highs group’s green technology innovation (Gti) is significantly higher than that of Lows.

Finally, we analyzed different profitability values and determined that, on average, the Highs group has a lower level of government subsidy intensity than the Lows group, suggesting that Lows group companies are more easily funded. From the perspective of environmental benefit, the environmental benefit of the Lows group is better. On an average Gti basis, the Highs group has significantly higher green technology innovation value (Gti) than the Lows group.

### 3.4. Stability Test of Variables

Here, ADF method is used to test the element root of each variable, and the results are shown in Table 3:

Table 3 shows that under the condition of 5% significance, the undeniable existence of unit root is demonstrated when the government’s low-carbon subsidy (Sub), green technology innovation (Gti), environmental performance (EP), enterprise size (Aversize), asset growth (Grow), profitability (Nsale) and other sequences are tested. However, the results of testing the first-order difference of each period sequence are contrary to the basic assumption that there is a unit root. It follows that all time series are unstable.

### 3.5. Co-Linearity Test

The model in this paper contains several variables, so the problem of multicollinearity needs to be tested, and here the variance inflation factor is proposed to be diagnosed. The specific diagnostic results are shown in Table 4.

By calculating the VIF (variance inflation factor) values of each independent variable of the linear panel model, it can be seen that the VIF values of all the independent variables are within 3.5. When the amount of data is sufficient, VIF < 10 and is considered to be free from severe multicollinearity, which indicates that there is no multicollinearity among the independent variables of the stepwise regression model and the model is reasonably reliable.

## 4. Empirical Analysis

Government subsidies may have different effects on companies with different sizes, asset growth and profitability. This paper classifies companies with different sizes, asset growth and profitability through the analysis of the whole sample. On the one hand, the average size of a company (size heterogeneous grouping variable) reflects the development degree of a company in the past; on the other hand, it is also the most recognizable characteristic of an enterprise in different life cycles. The asset growth (the heterogeneous grouping variable of asset growth) can better reflect the potential value of the company value. Profitability (the heterogeneous grouping variable of profitability) is the main indicator of the company’s future development. To put it simply, the three groups of variables are classified according to the past and future development situations, and their mutual relations are explored, and exploratory research is carried out in the aspects of goal setting, goal realization path, resource internalization path, resource allocation angle and so on.

### 4.1. GMM Estimation of Firm Size Heterogeneity

Based on the heterogeneity of company size, the estimated GMM values for the two types of Lows and Highs are shown in Table 5.

By comparing the GMM estimates of the three models, the following conclusions are drawn: First, with current Sub as the variable, the Lows group only has one Sub which has a significant positive effect on its own value. This indicates that, compared with large companies, government subsidies for small enterprises are more sustainable, that is, the higher the intensity of government subsidies received in the early stage, the easier it is to obtain high-intensity research subsidies in the current period. In addition, regardless of the company’s size, early profitability and environmental performance were not important factors in the current period of government subsidies.

Second, when Gti is taken as a variable, all the variables in the previous stage have a significant positive effect on Gti. The positive effect of Highs on Gti is not obvious. However, EP and Gti have obvious positive influences on the Gti of this period. In the Lows group, the influence of early Gti and Sub on current Gti is not obvious, while the influence of EP on Gti is obviously greater than that of the Highs group. As can be seen from the above table, the green technology innovation ability of large enterprises is relatively sustainable, and the environmental performance in the previous stage can also promote the improvement of their green technology innovation ability. In small enterprises, the environmental performance in the previous stage has a significant positive impact on the company’s green technology innovation ability. Among them, none of the government subsidies will improve our green technology innovation ability.

Finally, with the current EP as the dependent variable, all the three variables in the last stage produced a significant positive effect on environmental performance, while in the Highs group, the company’s early EP had no significant positive effect on the current period. Both Gti and Sub of the previous stage had a higher positive effect on the current EP than the Lows group. In the Lows group, early Sub had no significant positive effect on EP, but previous EP and Gti had significant positive effect on EP.

The above results show that, on the whole, the larger the government subsidy, the stronger the current environmental performance and technological innovation ability, the more obvious the improvement effect on environmental benefits. In other words, the government subsidy can improve the company’s green technology innovation ability and environmental performance, and the impact on the latter is more obvious. Large companies are efficiently using and rationally allocating external resources (government subsidies) and internal resources (green technology innovation) to promote enterprise development; small companies, on the other hand, have shown greater sustainability in their growth drive and a greater focus on using their own internal resources (green technologies) to improve environmental performance.

### 4.2. GMM Estimation of Heterogeneity of Corporate Asset Growth

According to the heterogeneity of the company’s asset growth, the estimated GMM values for the two types of Lows and Highs are shown in Table 6.

### 4.3. GMM Estimation of Corporate Profitability Heterogeneity

Based on the heterogeneity of corporate profitability, the estimated GMM values of the Lows and Highs groups are shown in Table 7.

Since detailed analysis was carried out in the the above section, only the main conclusions of the asset growth group and the profitability group are analyzed. It can be seen that last EP had a promoting effect on the increase in current Gti, and the Lows group was better than the Highs group. While the continuance coefficient and significance degree of EP in the Lows group were better than those of the Highs, the positive influence of Sub on EP also showed better performance in the Lows group. As for Sub, although Sub favored the Lows group, the Highs group was more likely to experience continuous research subsidy expenses. The Highs group had a positive effect on current Sub, while the Lows group did not. In the Highs group, early Sub had a positive effect on current Gti, and the Highs group had a large positive effect on current EP, which was higher than the average value, while the Lows group had no such effect. The results show that the early stage Sub of the Highs group had a significant positive correlation with the current Sub, Gti and EP. The Lows group’s early Sub only had a significant positive effect on the current EP. In addition, only in terms of the number of correlations on the significance test, the interaction between the three core variables in the Highs group is significantly higher than that of the Lows group, where only the effects of the previous EP on Gti and the effects of the previous Sub and EP on the current EP are present.

### 4.4. Robustness Tests

To ensure the accuracy and reliability of the results of the empirical analysis, robustness tests were conducted in this research by introducing the lagged period of the dependent variable as the independent variable and also using the estimation method of GMM. The detailed results are shown in Table 8.

The results from the Lows and Highs groups show that the government policy of low-carbon subsidies has a significant effect on the health of emerging firms, which is consistent with the previous results. Therefore, the model can be considered robust. Meanwhile, the problem of endogeneity is somewhat mitigated by the introduction of a one-period lag of the dependent variable in the model, i.e., explaining the next-period variables with the prior period variables, and the estimation method of the model is GMM, which can be used to control for model endogeneity. Therefore, from both perspectives, the endogeneity problem is mitigated, and since the results of this model are consistent with the results of the previous model, it can be concluded that the previous model is plausible.

## 5. Conclusions and Recommendations

### 5.1. Conclusions

This paper mainly discusses the interaction effect between the government’s low-carbon subsidies and the growth ability of green and low-carbon emerging industries so as to verify said effect. Overall, this paper studies the interaction between the intensity of low-carbon subsidies and the growth ability of new enterprises.

In general, the intensity of government subsidies in the first stage has a certain promoting effect on the company’s current profitability and asset growth, and the improvement effect on the former is more obvious. This shows that our government’s policy of low-carbon subsidies has a significant influence on the healthy development of emerging enterprises, in particular on accelerating the growth of enterprises, and is of great significance to the economic growth of our country.

In large-scale companies, the asset growth ability and profitability in the early stage have a significant positive impact on the current earnings, and the contribution of profitability to the company’s own performance is far greater than the average. For smaller companies, the asset growth ability and profitability in the early stage have a significant positive impact on the current earnings, and the asset growth ability has a far greater impact on the company’s own performance than the average. This shows that, from the perspective of the life cycle of a company, large companies have the characteristics of mature enterprises and focus more stable profits. Therefore, while small enterprises have the growth and rapid development as their main objective, industrial policies should focus on encouraging their rapid development.

The Highs of the early period government subsidy intensity, the current period government subsidy intensity, corporate asset growth ability and profitability have obvious positive correlation. The intensity of government subsidy in the early period of the Lows group only has a significant positive impact on the current asset growth ability. In addition, the interaction between the three core variables in the Highs group was also significantly higher than that of the Lows group. The Highs Highs group is characterized by the strength of multi-channel access to external resources (government subsidies), while the Lows Highs group is characterized by the strength of internalizing external resources through a single channel. The Highs Highs group is more efficient at allocating and utilizing resources from a systematic and holistic perspective. The Lows group’s understanding and utilization ability of external resources is not strong, so industrial policies can be adopted to guide it.

### 5.2. Policy Recommendations

#### 5.2.1. Advice to the Government

(1) Intensify the publicity of environmental awareness. This can be achieved, for example, by putting up more environmental protection propaganda slogans in public spaces, playing environmental protection propaganda videos on public LED screens, carrying out environmental education in schools, organizing students to participate in environmental protection initiatives and employing other aspects of publicity. Overall, the objective is to enhance the awareness of enterprises and consumers in regard to environmental protection so that they can deeply understand that environmental damage is closely related to their lives. Therefore, initiative should be taken to participate in various environmental protection activities, such as active promotion of the construction of green supply chain rather than outsider observation and evaluation of environmental policy.

(2) Give incentives to new green and low-carbon companies. In addition to financial subsidies, there are many ways to do so such as tax breaks for new energy vehicle enterprises, technical support for green and low-carbon enterprises, and cooperation between government and enterprise. Encourage purchase of raw materials through government channels to reduce costs, and offer government support for green and low-carbon emerging enterprises through its own publicity. The objective is to remove some obstacles for new companies to start their own businesses.

(3) Strengthen environmental management. The government should strengthen the management of environmental protection and strictly implement relevant laws and regulations. For those green and low-carbon enterprises that do not regulate production according to the requirements, corresponding penalties should be enforced, such as increasing the consumption tax rate of enterprises and depriving them of preferential treatment for government purchases. At the same time, by educating these companies about the harm they inflict, guilt can be invoked and the companies might start to promote the concept of low-carbon economy and sustainable development.

(4) Improve the formulation of relevant policies and regulations. At present, our country’s environmental protection baseline is still relatively low, and the relevant environmental regulations have not been strictly implemented. Many illegal behaviors have become a kind of default. Therefore, the environmental standard of our country must be promoted as soon as possible and the relevant regulations should be constantly improving. Specifically, environmental requirements should be refined to adhere to the world standard level.

(5) Encourage he participation and supervision of the media. News media, as a mass media, can not only guide the public, but also help the government to supervise enterprises. Improve consumers’ preference for low-carbon consumption and promote enterprises’ energy saving, emission reduction and environmental protection in order to reduce the government’s pressure on the environment to achieve the effect of proverbial killing of two birds with one stone. In all, the government should vigorously advocate for the participation of mass media.

#### 5.2.2. Advice to Green Start-Ups

(1) In order to promote low-carbon environment, create an environmental protection company image. Within the company, a good environment protection environment should be established, low-carbon concept inculcated to employees, low-carbon corporate culture advocated for, and the enterprise’s environmental protection awareness and moral concept should be enhanced. In this way, companies can fully consider environmental benefits in all decision-making processes and achieve low-carbon collaboration and management in their supply chains. In addition, a good corporate image is also conducive to improving the image of enterprises in the eyes of consumers so as to promote the competition of enterprises in the market to some extent.

(2) Vigorously develop low-carbon technologies and new energy technologies. Although the research and optimization of new energy technology entails huge costs, reducing the company’s profits, in the long run, with the development of low-carbon economy, along with the development of technology and society, traditional energy vehicles will be gradually phased out. Therefore, research and development of new energy technology is the core competence of a company to conquer the market. Through energy saving and emission reduction, improving the endurance capacity of electric vehicles, accelerating charging and other new energy vehicles and improving various supporting infrastructure it is possible to reduce the cost of mass production in the future.

(3) Maintain good communication and cooperation with upstream and downstream suppliers. Regardless of circumstances, be it daily life, study, business, or even war, suppliers play an irreplaceable role. In business competition, an enterprise’s competitors are manufacturers of similar products, and suppliers and retailers usually do not have conflicts of interest. Therefore, it is necessary to strengthen communication with upstream enterprises and establish a good partnership, which will be of great help to the future development of the company. In addition, in order to build a green and low-carbon supply chain, cooperation in the supply chain is needed. Otherwise, if only the manufacturing enterprises are saving energy and reducing emissions, the suppliers are still operating in the traditional supply chain mode, which may lead to losses.

(4) Strictly implement the state environmental protection policy. The policies formulated by the government are not only the development direction of a country, but also a beacon for a company. Enterprises should carefully study the policies related to environmental protection, clarify the purpose of the government, and strictly abide by the environmental protection policies. At the same time, it will not harm the interests of enterprises; on the contrary, this will help enterprises find more market opportunities.

## Figures and Tables

**Table 1:** Summary of variables.

Variable	Encoding of Variables	Method of Measurement	Type of Variable
Intensity of government subsidy	Sub	Government subsidy/total assets ×100%	Core variable
Green technology innovation	Gti	Natural logarithm of R&D investment	Core variable
Environmental protection performance	EP	Environmental protection input/operating income	Core variable
Size of enterprise	Aversize	The average company size from 2014 to 2021 based on natural logarithm and the range divided by median. The enterprise group below the median value is the relatively small enterprise group (Lows group), otherwise it is called a larger enterprise group (Highs group)	Firm size is a heterogeneous grouping variable
Growth in assets	Grow	The company’s average revenue growth from 2014 to 2021. With the median value as the range, the number of companies below the median is in Lows group, and the opposite is a relatively large company (Highs group)	Heterogeneous grouping variable of asset growth
Profitability	Nsale	The average earnings value of the company from 2014 to 2021 based on the natural logarithm. The range is divided by the median. Those below the median are relatively small companies(Lows group), otherwise they are relatively large companies (Highs group)	Profitability is a heterogeneous grouping variable

**Table 2:** Descriptive statistics of variables.

Category		Variable	Mean	Sd	Min	Max
**Full sample**		Sub	0.463	0.509	0.000	4.835
		Gti	17.13	4.842	0.000	22.236
		EP	0.0014	0.0012	0.0001	0.0052
		Aversize	22.36	1.088	20.263	26.529
		Grow	0.392	3.487	−0.494	109.27
		Nsale	0.069	0.412	−8.269	1.211
Heterogeneity of scale	Highs	Sub	0.433	0.491	0	3.627
		Gti	16.74	4.826	3.456	21.364
		EP	0.0004	0.0010	0	0.0041
		Aversize	23.679	0.872	22.592	27.070
	Lows	Sub	0.478	0.529	0.000	4.835
		Gti	17.69	4.926	0	18.657
		EP	0.0023	0.0023	0.0001	0.0061
		Aversize	21.947	0.476	20.263	22.564
Heterogeneity of asset growth	Highs	Sub	0.445	0.534	0	4.835
		Gti	16.86	4.927	2.367	22.236
		EP	0.0010	0.0021	0	0.0052
		Grow	1.387	6.667	0.231	54.234
	Lows	Sub	0.485	0.481	0	4.158
		Gti	17.26	4.726	0	22.692
		EP	0.0021	0.0009	0.0001	0.0053
		Grow	0.102	0.131	−0.137	0.238
Heterogeneity of profitability	Highs	Sub	0.352	0.457	0.087	4.376
		Gti	15.67	3.619	2.341	20.341
		EP	0.0008	0.0006	0.0001	0.0050
		Nsale	1.254	6.027	0.620	51.061
	Lows	Sub	0.229	0.261	0.091	4.376
		Gti	17.22	4.849	0	22.236
		EP	0.0016	0.0022	0.001	0.0049
		Nsale	0.426	5.819	−0.268	0.361

**Table 3:** Results of stability of variables.

	ADF Value	Pvalues	Isitsmooth		ADF Value	*p* Values	Isitsmooth
Sub	−2.26	0.18	No	D (Sub)	−8.24	0.00	Is
Gti	−2.01	0.24	No	D (Gti)	−8.01	0.00	Is
EP	−1.37	0.54	No	D (EP)	−7.26	0.00	Is
Aversize	−2.03	0.27	No	D (Aversize)	−9.92	0.00	Is
Grow	−2.03	0.27	No	D (Grow)	−10.03	0.00	Is
Nsale	−1.25	0.89	No	D (Nsale)	−6.09	0.00	Is

Note: (1) The optimal lag order, according to Modified AIC information standard, has a maximum lag time of 7 stages. (2) The format of the variable is that with a constant term.

**Table 4:** Table of variance inflation factors.

Variables	Lows	Highs
Sub	1.57	2.54
Gti	1.97	2.58
EP	1.64	3.24
Aversize	3.25	2.24
Grow	3.07	1.03
Nsale	1.47	1.20
Mean VIF	1.24	1.35

**Table 5:** Summary of GMM estimation results of PVAR model based on enterprise size heterogeneity.

	(1) Lows		(2) Highs	
**h_sub**				
L.h_sub	0.236 ***	(2.377)	0.162	(0.643)
L.h_Gti	0.118	(0.877)	0.274	(1.183)
L.h_EP	0.081	(1.132)	−0.014	(−0.245)
h_Gti				
L.h_sub	0.037	(1.52)	0.079	(1.561)
L.h_Gti	−0.003	(−0.041)	0.386 **	(2.264)
L.h_EP	0.093 ***	(4.396)	0.029 **	(2.132)
h_EP				
L.h_sub	0.123	(1.591)	0.619 *	(1.887)
L.h_Gti	0.442 ***	(2.887)	0.976 **	(2.162)
L.h_EP	0.164 ***	(3.682)	0.137	(1.53)
N	397		397	
AIC	2.541		2.522	
BIC	6.694		6.349	
HQIC	4.187		3.963	

Note: In the table, L. represents an advanced period, and h_ indicates that a variable has been positively averaged. t statistics in parentheses, * *p* < 0.1, ** *p* < 0.05, *** *p* < 0.01.

**Table 6:** Summary of GMM estimation results of PVAR model based on heterogeneity of enterprise asset growth.

	(1) Lows		(2) Highs	
**h_sub**				
L.h_sub	0.224	(1)	0.225 **	(2.295)
L.h_Gti	−0.247	(−1.306)	0.345 **	(2.275)
L.h_EP	0.023	(0.275)	0.018	(0.3260
h_Gti				
L.h_sub	0.023	(0.53)	0.047 **	(2.173)
L.h_Gti	0.185	(1.295)	0.166	(1.285)
L.h_EP	0.08 ***	(3.203)	0.039 ***	(3.346)
h_EP				
L.h_sub	0.32 **	(2.601)	0.224 *	(1.795)
L.h_Gti	0.344	(1.673)	0.775 **	(2.519)
L.h_EP	0.147 ***	(3.08)	0.126 *	(1.775)
N	397		397	
AIC	1.115		3.475	
BIC	5.267		7.631	
HQIC	2.761		5.122	

Note: In the table, L. represents an advanced period, and h_ indicates that a variable has been positively averaged. t statistics in parentheses, * *p* < 0.1, ** *p* < 0.05, *** *p* < 0.01.

**Table 7:** Summary of GMM estimation results of PVAR model based on corporate profitability heterogeneity.

	(1) Lows		(2) Highs	
**h_sub**				
L.h_sub	0.115	(0.84)	0.267 **	(2.34)
L.h_Gti	−0.183	(−1.04)	0.309 **	(2.09)
L.h_EP	0.031	(0.35)	0.021	(0.48)
h_Gti				
L.h_sub	0.036	(0.68)	0.057 **	(2.26)
L.h_Gti	0.171	(1.31)	0.174	(1.05)
L.h_EP	0.052 ***	(3.09)	0.044 ***	(3.15)
h_EP				
L.h_sub	0.339 **	(2.76)	0.235 *	(1.58)
L.h_Gti	0.281	(1.37)	0.731 **	(2.59)
L.h_EP	0.195 ***	(3.31)	0.125 *	(1.63)
N	397		397	
AIC	1.078		3.304	
BIC	5.152		7.454	
HQIC	2.671		5.035	

Note: In the table, L. represents an advanced period, and h_ indicates that a variable has been positively averaged. t statistics in parentheses, * *p* < 0.1, ** *p* < 0.05, *** *p* < 0.01.

**Table 8:** Robustness tests.

Variables	Lows GMM	Highs GMM
Sub	0.000942 (0.04)	0.0164 (0.55)
Gti	−0.392 *** (−5.47)	−0.626*** (−6.01)
EP	0.763 ***(18.37)	0.799 (16.99)
Aversize	0.00202 (1.24)	0.00125 (0.79)
Grow	0.347 *** (25.41)	0.352 *** (25.15)
Nsale	0.0223 ** (7.35)	0.0221 * (7.27)
Constants	0.939 *** (15.22)	0.897 *** (14.71)
AR (1)	0.062	0.024
AR (2)	0.859	0.583
Sargan	1163.2	1195.1
N	1231	1231

Note: *** has significance at 1%, ** has significance at 5%, * has significance at 10%.

## Data Availability

The data are not publicly available due to privacy restrictions.
